# Efficacy of Low-Dose Aspirin in Colorectal Cancer Risk Prevention is Dependent on *ADH1B* and *ALDH2* Genotype in Japanese Familial Adenomatous Polyposis Patients

**DOI:** 10.1158/2767-9764.CRC-22-0088

**Published:** 2022-06-23

**Authors:** Kanae Mure, Hideki Ishikawa, Michihiro Mutoh, Mano Horinaka, Takahiro Otani, Sadao Suzuki, Keiji Wakabayashi, Toshiyuki Sakai, J-FAPP Study IV group, Yasushi Sato, Hisashi Doyama, Masahiro Tajika, Shinji Tanaka, Takahiro Horimatsu, Yoji Takeuchi, Hiroshi Kashida, Jun Tashiro, Yasumasa Ezoe, Takeshi Nakajima, Hiroaki Ikematsu, Shinichiro Hori, Tetsuji Takayama, Yoshio Ohda

**Affiliations:** 1 Department of Public Health, Wakayama Medical University School of Medicine, Wakayama, Japan.; 2 Department of Molecular-Targeting Prevention, Kyoto Prefectural University of Medicine, Kyoto, Japan.; 3 Ishikawa Gastroenterology Clinic, Osaka, Japan.; 4 Epidemiology and Prevention Group, Research Center for Cancer Prevention and Screening/Center for Public Health Sciences, National Cancer Center, Tokyo, Japan.; 5 Department of Drug Discovery Medicine, Kyoto Prefectural University of Medicine, Kyoto, Japan.; 6 Department of Public Health, Nagoya City University Graduate School of Medical Sciences, Aichi, Japan.; 7 Graduate Division of Nutritional and Environmental Sciences, University of Shizuoka, Shizuoka, Japan.; * All group members are considered authors. Group members' names and affiliations are listed in the Acknowledgments.

## Abstract

Aspirin has gained great attention as a cancer preventive agent. Our previous study revealed that the low-dose aspirin prevents colorectal tumor recurrence in Japanese patients with colorectal adenomas and/or adenocarcinomas, whereas aspirin increases risks in smokers and has no effects on regular drinkers. Our recent study revealed that aspirin reduces polyp growth in Japanese patients with familial adenomatous polyposis (FAP). In this study, we have studied the association of genotypes of alcohol metabolizing enzymes (ADH1B and ALDH2) on aspirin's efficacy of suppressing polyp growth (≥5 mm) in a total of 81 Japanese patients with FAP. Our study revealed that aspirin showed significant preventive effects for patients with *ADH1B*-AA and AA+GA types [OR = 0.21; 95% confidence interval (CI), 0.05–0.95, and OR = 0.31; 95% CI, 0.10–0.95, respectively], and for patients with *ALDH2*-GG and GG+GA types (OR = 0.10; 95% CI, 0.01–0.92, and OR = 0.29; 95% CI, 0.09–0.94, respectively), but not for patients with *ADH1B*-GG and GA+GG types, and *ALDH2*-AA and GA+AA types. In addition, substantial preventive effects of aspirin were seen for patients with *ADH1B*-AA type who do not drink regularly (<3 times/week, OR = 0.11; 95% CI, 0.02–0.78), where a statistically significant interaction between aspirin and *ADH1B* was observed (*P*_interaction_ = 0.036). Results from this exploratory study strongly indicate that aspirin is beneficial in prevention of polyp growth for patients with FAP with *ADH1B*-AA and AA+GA types, and *ALDH2*-GG and GG+GA types. Taken together, we propose *ADH1B* and *ALDH2* as candidate markers for the personalized prevention by aspirin.

**Significance:**

Aspirin is beneficial to patients with FAP with *ADH1B*-AA and AA+GA types or *ALDH2*-GG and GG+GA types. *ADH1B* and *ALDH2* genotypes can be the markers for the personalized prevention of colorectal cancer by aspirin.

## Introduction

Aspirin has gained great attention as a cancer preventive agent. Recently, we reported that low-dose aspirin prevents colorectal polyp growth in Japanese patients with familial adenomatous polyposis (FAP) without a history of colectomy. We conducted a randomized double-blind, placebo-controlled trial with a 2 × 2 factorial design to determine the individual and concerted effects of low-dose aspirin and mesalazine, a NSAID (J-FAPP Study IV). Aspirin has prevented polyp growth (OR = 0.37; 95% confidence interval (CI), 0.16–0.86] but mesalazine showed no effect (1). FAP is an autosomal dominant syndrome primarily caused by germline mutations in *adenomatous polyposis coli* (*APC*). Somatic mutations in APC have been observed in 80% of colorectal adenomas and carcinomas, therefore FAP has been considered as a model for colorectal cancer (2). FAP and sporadic colorectal cancer share risk factors such as genetic alterations and lifestyle factors (e.g., smoking and heavy alcohol drinking). Our previous study revealed that the low-dose aspirin prevents recurrence of colorectal tumor in Japanese patients with colorectal adenoma and/or adenocarcinomas (J-CAPP Study; ref. 3). In addition, aspirin increases polyp recurrence risks in smokers but has no effects on regular drinkers (≥3 times/week). Ethanol is oxidized by alcohol dehydrogenase 1B (ADH1B) to produce acetaldehyde, and acetaldehyde is further oxidized to acetate by aldehyde dehydrogenase 2 (ALDH2; ref. 4). A previous study has been shown that the enzymatic activities of ADH1B and ALDH2 are influenced by genotypes of *ADH1B* (rs1229984, A/G) and *ALDH2* (rs671, G/A; ref. 5). *ADH1B*-AA rapidly metabolizes ethanol to acetaldehyde, whereas *ADH1B*-GG metabolizes slowly. *ALDH2*-GG metabolizes acetaldehyde, but *ALDH2*-AA is inactive. Interestingly, *ADH1B*-AA+GA and *ALDH2*-GA+AA genotypes are exclusively found in eastern Asian populations and are related to the frequency of upper digestive cancer (6). To date, the relationships of these genotypes and colorectal cancer have not been established.

In this study, we have examined the correlation of *ADH1B* (rs1229984) and *ALDH2* (rs671) genotypes with the aspirin's efficacy on preventing polyp growth in the patients with FAP, where their drinking status was also considered. Several genetic variants that affect the efficacy of aspirin have been reviewed previously (7, 8). However, there has been no study that investigates the effects of *ADH1B* or *ALDH2* genotypes on aspirin's efficacy.

## Materials and Methods

### Trial Design and Patients’ Description

Patients were rerecruited from the single clinic which attended to the previous multicenter (*n* = 11; located throughout Japan), randomized, double-blind, placebo-controlled clinical trial used a 2 × 2 factorial design (J-FAPP Study IV; ref. 1). Details of trials of J-FAPP Study IV was described previously (1). Briefly, the effects of administrating low-dose enteric-coated aspirin tablets (Bayaspirin, 100 mg/day) and/or mesalazine (Pentasa, 2 g/day) for 8 months were evaluated on inhibiting the growth of colorectal polyps in Japanese patients with FAP. Colorectal polyps (≥5 mm) were removed endoscopically prior to the trial. Patients took low-dose enteric-coated aspirin tablets (100 mg/tablet) and their placebo counterparts (Bayer Yakuhin, Ltd.) and/or mesalazine tablets (250 mg/tablet) and their placebo counterparts (Kyorin Pharmaceutical Co., Ltd.) until 1 week before the 8-month colonoscopy.

Polyps in ≥5.0 mm size that were detected as twice the diameter of the polypectomy snare (ZEMEX Co.) was removed and collected during colonoscopy for histologic examination. Patients with uncurable cancer; taking antithrombotic or anticoagulant agents; a history of stroke, including transient ischemic attack; and other diseases were excluded from the J-FAPP Study IV. Among patients who participated in the J-FAPP Study IV, all patients belonged to the single-center clinic were rerecruited and provided written informed consent prior to this study. This study followed the principles stated in the Declaration of Helsinki and was approved by the ethical committees for Analytical Research on the Human Genome of Wakayama Medical University (approval no. 117).

### Questionnaire and Genotyping

Patients were requested to provide information such as height, body weight, medical history, smoking status, alcohol consumption, and intake of any NSAID prior to the J-FAPP Study IV. The smoking habits were categorized into two groups (yes: currently smoking, no: never and formerly smoking). Alcohol drinking habits were categorized into two groups (regularly drinking: ≥3 times per week, nonregularly drinking: otherwise).

Venous blood was collected in a heparinized vacuum blood collection tube and blotted onto a Whatman FTA card (FTA elute microcard, GE Healthcare UK Limited). Genomic DNA was extracted by using DNA Extract All Reagents Kit (Thermo Fisher Scientific) from the 2 mm punched out FTA sample. TaqMan SNP Assays used in this study were purchased from Thermo Fisher Scientific (*ADH1B*, rs1229984, C_2688467_20, *ALDH2*, rs671, C_11703892_10). Genotypes of *ADH1B* and *ALDH2* were examined on Step One Plus Real-Time PCR systems (Applied Biosystems).

### Statistical Analysis

Statistical analyses were performed using R version 4.0.3 (9). Differences in age among four groups were analyzed by one-way ANOVA. Differences in age between administrating aspirin and placebo groups were analyzed by Student *t* test. Differences in the categorical variables such as sex, alcohol drinking, and smoking habits were analyzed by Fisher exact test. The effects of aspirin on polyp growth were analyzed by logistic regression analyses adjusted for age, sex, alcohol drinking and smoking habits, and mesalazine intake. *ADH1B*-GA+GG and *ALDH2*-GA+AA were used as the dominant model, and *ADH1B*-AA+GA and *ALDH2*-GG+GA were used as the recessive model. Multiplicative interactions between aspirin and *ADH1B* or *ALDH2* genotypes were assessed in the logistic regression analyses. *P* values of <0.05 were considered statistically significant.

### Data Availability

The data generated in this study are available upon reasonable request from the corresponding author.

## Results

The basic characteristics of patients in this study are summarized in Table 1. There were no significant differences among groups, except a significant difference was seen in the distribution of regular drinking among the *ALDH2* genotype. The majority of *ALDH2*-GA+AA types do not drink alcoholic beverages regularly (Supplementary Table S1).

**TABLE 1:** Base line characteristics of the patients.

	Placebo: Placebo	Aspirin: Placebo	Placebo: Mesalazine	Aspirin: Mesalazine	*P* ^b^	Placebo	Aspirin	*P* ^c^
*N*	19	18	22	22		41	40	
Age (years), mean (SD)	33.2 (8.5)	38.8 (12.6)	36.8 (9.1)	35.6 (11.5)	0.424	35.1 (8.9)	37.1 (12.0)	0.401
Sex, male (%)	10 (52.6)	10 (55.6)	11 (50.0)	12 (54.6)	0.990	21 (51.2)	22 (55.0)	0.825
Regularly drinking, yes (%)^a^	5 (26.3)	5 (27.8)	3 (13.6)	3 (13.6)	0.536	8 (19.5)	8 (20.0)	1.000
Smoking	3 (15.8)	2 (11.1)	1 (4.6)	1 (4.6)	0.549	4 (9.8)	3 (7.5)	1.000
*ADH1B*								
AA	15 (79.0)	13 (72.2)	15 (68.2)	13 (59.1)	0.396	30 (73.2)	26 (65.0)	0.729
GA	3 (15.8)	4 (22.2)	7 (31.8)	9 (40.9)		10 (24.4)	13 (32.5)	
GG	1 (5.3)	1 (5.6)	0 (0.0)	0 (0.0)		1 (2.4)	1 (2.5)	
Dominant, GA+GG	4 (21.1)	5 (27.8)	7 (31.8)	9 (40.9)	0.567	11 (26.8)	14 (35.0)	0.477
Recessive, AA+GA	18 (94.7)	17 (94.4)	22 (100.0)	22 (100.0)	0.348	40 (97.6)	39 (97.5)	1.000
*ALDH2*								
GG	9 (47.4)	8 (44.4)	14 (63.6)	11 (50.0)	0.580	23 (56.1)	19 (47.5)	0.266
GA	9 (47.4)	7 (38.9)	8 (36.4)	9 (40.9)		17 (41.5)	16 (40.0)	
AA	1 (5.3)	3 (16.7)	0 (0.0)	2 (9.1)		1 (2.4)	5 (12.5)	
Dominant, GA+AA	10 (52.6)	10 (55.6)	8 (36.4)	11 (50.0)	0.621	18 (43.9)	21 (52.5)	0.508
Recessive, GG+GA	18 (94.7)	15 (83.3)	22 (100.0)	20 (90.9)	0.220	40 (97.6)	35 (87.5)	0.109

NOTE: *N* (%).

^a^Drink alcoholic beverage ≥3 times per week.

^b^ *P*: Fisher exact test for categorical variables, and one-way ANOVA for continuous variables.

^c^ *P*: Fisher exact test for categorical variables, and *t* test for continuous variables.

The logistic regression analyses (adjusted for age, sex, alcohol drinking, smoking, mesalazine intake, and *ADH1B* and *ALDH2* genotypes in the additive and recessive models) indicate that aspirin intake correlates with significantly reduced risks of poly growth (OR = 0.29; 95% CI, 0.09–0.89; as shown in Table 2). *ALDH2* genotype showed increased risk in the additive model (OR = 2.62; 95% CI, 1.05–6.50). When drinking status was considered, aspirin intake showed no effects in the nonregular drinkers in any models. *ALDH2* genotype showed increased risk in the additive model (OR = 2.93; 95% CI, 1.12–7.71). Significant interaction between aspirin and *ADH1B* genotype was observed in patients who do not drink regularly (*P*_interaction_ = 0.036). Detail results of all covariates were presented in Supplementary Table S2. Aspirin intake and *ADH1B* or *ALDH2* genotypes showed no significant effects in regular drinkers (Supplementary Table S3).

**TABLE 2:** Effects of aspirin, and *ADH1B* and *ALDH2* genotypes on suppressing polyp growth after intervention.

**(a) Additive model**						
	**All**			**Nonregular drinkers^a^**		
	**OR^b^**	**95% CI**	***P*_int_ ^c^**	**OR**	**95% CI**	***P*_int_**
Aspirin	**0.29**	**0.09–0.89**		0.35	0.09–1.29	
*ADH1B*	1.51	0.57–3.99	0.664	1.09	0.32–3.69	**0.036**
*ALDH2*	**2.62**	**1.05–6.50**	0.184	**2.93**	**1.12–7.71**	0.181
**(b) Dominant model**						
	**All**			**Nonregular drinkers**		
	**OR**	**95% CI**	***P*_int_**	**OR**	**95% CI**	***P*_int_**
Aspirin	0.36	0.12–1.04		0.46	0.13–1.55	
*ADH1B*-GA+GG	1.14	0.37–3.52	0.596	0.81	0.20–3.23	0.054
*ALDH2*-GA+AA	2.18	0.72–6.66	0.099	2.60	0.78–8.70	0.108
**(c) Recessive model**						
	**All**			**Nonregular drinkers**		
	**OR**	**95% CI**	***P*_int_**	**OR**	**95% CI**	***P*_int_**
Aspirin	**0.29**	**0.09–0.92**		0.31	0.08–1.16	
*ADH1B*-AA+GA	NA			NA		
*ALDH2*-GG+GA	0.14	0.02–1.19	0.143	0.15	0.02–1.30	0.111

^a^Drink alcoholic beverage <3 times/week.

^b^OR: odds ratio adjusted for age, sex, drinking and smoking habits, and mesalazine intake.

^c^Multiplicative interaction of aspirin and *ADH1B* or *ALDH2* genotypes were assessed in the logistic regression analyses.

**FIGURE 1:**
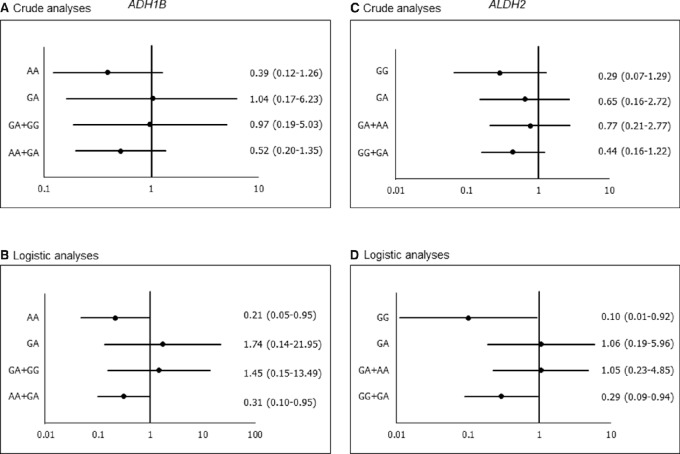
Effects of *ADH1B* and *ALDH2* genotypes on the aspirin's effects on suppressing growth of polyps 5 mm or larger. Crude ORs and 95% CIs for *ADH1B* genotypes (**A**), adjusted ORs and 95% CIs for *ADH1B* genotypes (**B**), crude ORs and 95% CIs for *ALDH2* genotypes (**C**), adjusted ORs and 95% CIs for *ALDH2* genotypes (**D**). Numbers appearing on the graph represent OR (black dot) and the 95% CI (bars). Adjusted ORs were estimated with adjustment for age, sex, alcohol drinking and smoking habits, and mesalazine intake in the logistic regression analyses. The *y*-axis is in logarithmic scale.

When the effects of aspirin intake were analyzed by genotypes, significant reducing risks in patients with *ADH1B*-AA and AA+GA types (OR = 0.21; 95% CI: 0.05–0.95, and OR = 0.31; 95% CI: 0.10–0.95, respectively) were observed in the multivariate logistic analyses (Fig. 1). In patients with *ALDH2*-GG and GG+GA types, aspirin intake was also correlated with significant reducing risks (OR = 0.10; 95% CI, 0.01–0.92, and OR = 0.29; 95% CI, 0.09–0.94, respec-tively).

Finally, subgroup analyses with regard to their drinking status were performed (Table 3). Aspirin intake showed significant reducing risks only in the *ADH1B*-AA type who do not drink alcoholic beverages regularly (OR = 0.11; 95% CI, 0.02–0.78).

**TABLE 3:** Effects of aspirin on suppressing polyp growth and *ADH1B* and *ALDH2* genotypes in nonregular drinkers (<3 times/week).

	No	Yes	Total	OR^a^	95% CI
Placebo	20	13	33	1	
Aspirin	23	9	32	0.35	0.09–1.29
**ADH1B-AA**					
	**No**	**Yes**	**Total**	**OR**	**95% CI**
Placebo	14	12	26		
Aspirin	16	4	20	**0.11**	**0.02–0.78**
***ADH1B-*GA+GG**					
	**No**	**Yes**	**Total**	**OR**	**95% CI**
Placebo	6	1	7		
Aspirin	7	5	12	3.50	0.25–48.55
***ALDH2-*GG**					
	**No**	**Yes**	**Total**	**OR**	**95% CI**
Placebo	11	5	16		
Aspirin	11	1	12	0.001	<0.001–6.01
***ALDH2-*GA+AA**					
	**No**	**Yes**	**Total**	**OR**	**95% CI**
Placebo	9	8	17		
Aspirin	12	8	20	1.05	0.23–4.85

^a^OR adjusted for age, sex, smoking habits, and mesalazine intake.

## Discussion

The direct association between aspirin and ADH1B and ALDH2 has only been seen as acute effects in *in vitro* study involving ethanol. After alcohol intake, aspirin inhibits ADH activities through noncompetitive fashion thereby increasing blood alcohol concentrations (10, 11), and ALDH2 through uncompetitive fashion (12). In this exploratory study, a significant preventive effect of aspirin was detected in patients with FAP with *ADH1B*-AA and AA+GA types, *ALDH2*-GG and GG+GA types, and *ADH1B*-AA type who do not drink regularly. To our knowledge, this is the first study that assessed the effects of *ADH1B* and *ALDH2* genotypes on efficacy of aspirin on suppressing polyp growth 5 mm or larger.

It is intriguing that a significant interaction between aspirin and *ADH1B* genotype was observed in patients who do not drink regularly, as well as aspirin showed a significant preventive effect on patients with *ADH1B*-AA without regularly drinking habit. These results imply that the aspirin's preventive effect is influenced by the *ADH1B* genotype most likely due to ADH1B metabolizing nontraditional substrates other than ethanol. Besides ethanol, ADH1B is known to oxidize endogenous aliphatic alcohol such as retinol and lipid peroxidation products that are associated with the development of colorectal cancer (13, 14). Acetylation of Lys331 and Lys340 of ADH1B protein has been detected in high frequency for colorectal tumors that are paired with liver metastasis (15). *ADH1B* is downregulated in colorectal cancer by *myc* (16), which is associated with hyperactivation of Wnt signaling. A lower expression of *ADH1B* at the mRNA level was also observed in adenomas compared with adjacent normal mucosa (14). A recent study indicated that ADH1B is involved in the metabolic activity of adipose tissues that is associated with insulin resistance. Furthermore, in that study, ADH1B is linked to progression of type 2 diabetes mellitus which is a risk factor for colorectal cancer (17). Taken together, these studies suggest that ADH1B plays an important role in the development of colorectal cancer in the absence of ethanol, that may relate to the aspirin's efficacy observed in our study.

In this study, significant effects of aspirin were also detected in patients with FAP with *ALDH2*-GG and GG+GA types, although *ALDH2* showed increased risk in the additive model. Aspirin is known to inhibit COX-1 to suppress the production of arachidonic acid, and also reduce the extent of the iron-induced oxidative stress and lipid peroxidation and prevent release of toxic aldehydes (e.g., malondialdehyde and 4-hydroxynoneal, 4-HNE; ref. 18). Besides acetaldehyde derived from ethanol, ALDH2 also metabolizes these toxic aldehydes (19). ALDH2 has shown to play a protective role in myocardial infarction via modulating the β-catenin/Wnt signaling (20). It is highly possible that both aspirin and ALDH2 orchestrate against β-catenin/Wnt signaling. In addition, *ALDH2*-GA+AA genotype, which is exclusively highly populated in Eastern Asia, seems to have an important role against polyp growth in patients with FAP. To our knowledge there are no studies that accessed the effects of *ALDH2* genotypes in patients with FAP to date. Further study is necessary to define the role of *ALDH2* genotype in polyp growth.

There are some limitations in this study. First, because the sample size is small, we have not been able to access the interaction between aspirin and *ADH1B* in the recessive model. We also have not been able to investigate the effects of alcohol drinking. There is a caveat that we have possible selection bias in this study. Second, the detail information about duration of smoking or regularly alcohol drinking and other cofounders that might affect result was not available. We are currently preparing for a larger number of patients who are warranted to clarify the gene–environment interaction.

In conclusion, patients with FAP with *ADH1B*-AA and AA+GA types, and *ALDH2*-GG and GG+GA types can get benefit of aspirin's preventive effects on suppressing polyp growth. In other words, *ADH1B* and *ALDH2* genotypes can be candidates for consideration of personalized prevention of colorectal cancer by aspirin.

## Supplementary Material

Supplementary Data Table S1, Table S2, Table S3
