# Supplementary material for: Efficacy of Low-Dose Aspirin in Colorectal Cancer Risk Prevention is Dependent on ADH1B and ALDH2 Genotype in Japanese Familial Adenomatous Polyposis Patients
Source: Cancer Res Commun. 2022 Jun 23;2(6):483–8. doi: 10.1158/2767-9764.CRC-22-0088 (PMC10010329; doi:10.1158/2767-9764.CRC-22-0088)
Supplement: Supplementary Data — Table S1, Table S2, Table S3 [file crc-22-0088-s01.pdf]

Supplemental TableS1. Distribution of *ADH1B* and *ALDH2* genotypes and their drinking status.

| | | All | | | Placebo | | | Aspirin | | |
|---|---|---|---|---|---|---|---|---|---|---|
| Regularly Drinking [1] | | No | Yes | P [2] | No | Yes | P | No | Yes | P |
| N | | 65 | 16 | | 33 | 8 | | 32 | 8 | |
| *ADH1B* | AA | 46 (70.8) | 10 (62.5) | 0.554 | 26 (78.8) | 4 (50.0) | 0.178 | 20 (62.5) | 6 (75.0) | 0.689 |
| | GA+GG | 19 (29.2) | 6 (37.5) | | 7 (21.2) | 4 (50.0) | | 12 (37.5) | 2 (25.0) | |
| *ALDH2* | GG | 28 (43.1) | 14 (87.5) | **0.002** | 16 (48.5) | 7 (87.5) | 0.059 | 12 (37.5) | 7 (87.5) | **0.017** |
| | GA+AA | 37 (56.9) | 2 (12.5) | | 17 (51.5) | 1 (12.5) | | 20 (62.5) | 1 (12.5) | |

N (%)

[1] Drinking alcoholic beverage ≥ 3 times per week.

[2] P: Fisher's exact test.

Supplemental Table S2. Effects of covariates used in the multivariate logistic analyses on polyp growth

| | | No | Yes | OR [2] | 95%CI |
|---|---|---|---|---|---|
| Sex | women | 25 | 13 | | |
| | men | 28 | 15 | 1.27 | 0.44-3.68 |
| Regularly drinking [1] | no | 43 | 22 | | |
| | yes | 10 | 6 | 1.59 | 0.38-6.64 |
| Current smoking | no | 47 | 27 | | |
| | yes | 6 | 1 | 0.11 | 0.01-1.37 |
| Mesalazine | no | 24 | 13 | | |
| | yes | 29 | 15 | 0.94 | 0.32-2.75 |

[1] Drink alcohol beverage ≥ 3 times per week.

[2] Odds ratio adjusted for aspirin, age, sex, smoking habits, mesalazine intake, *ADH1B* and *ALDH2* genotypes.

Supplemental Table S3. Effects of aspirin, and *ADH1B* and *ALDH2* genotypes on suppressing polyp growth after intervention in regular drinkers (≥ 3 days/week)

| | Additive model | | Dominant model | | Recessive model | |
|---|---|---|---|---|---|---|
| | OR [1)] | 95% CI | OR | 95% CI | OR | 95% CI |
| Aspirin | 0.15 | 0.002-10.56 | 0.13 | 0.002-10.34 | NA | |
| *ADH1B* | 5.41 | 0.29-100.51 | 6.08 | 0.21-176.68 | | |
| *ALDH2* | NA | | NA | | | |

[1)] OR: odds ratio adjusted for age, sex, drinking and smoking habits, and mesalazine intake.
